# A State-of-the-Art Review on the Wear of the Occlusal Surfaces of Natural Teeth and Prosthetic Crowns

**DOI:** 10.3390/ma13163525

**Published:** 2020-08-10

**Authors:** Ana Catarina Branco, Rogério Colaço, Célio Gabriel Figueiredo-Pina, Ana Paula Serro

**Affiliations:** 1 Centro de Química Estrutural (CQE), Instituto Superior Técnico, University of Lisbon, 1049-001 Lisbon, Portugal; ana.branco@tecnico.ulisboa.pt (A.C.B.); anapaula.serro@tecnico.ulisboa.pt (A.P.S.); 2 Centro de Desenvolvimento de Produto e Transferência de Tecnologia (CDP2T), Department of Mechanical Engineering, Instituto Politécnico de Setúbal, 2910-761 Setúbal, Portugal; 3 Centro de Investigação Interdisciplinar Egas Moniz (CiiEM), Instituto Universitário Egas Moniz, 2829-511 Caparica, Portugal; 4 Instituto de Engenharia Mecânica (IDMEC), Department of Mechanical Engineering, Instituto Superior Técnico, University of Lisbon, 1049-001 Lisbon, Portugal; rogerio.colaco@tecnico.ulisboa.pt; 5 Centro de Física e Engenharia de Materiais Avançados (CeFEMA), Instituto Superior Técnico, University of Lisbon, 1049-001 Lisbon, Portugal

**Keywords:** natural teeth, dental restorative materials, prosthetic dental mareials, biotribology, occlusal surfaces, wear mechanims

## Abstract

This review focuses on the wear mechanisms of natural and restorative dental materials, presenting a comprehensive description and analysis of the works published in the last two decades on the wear at the interface of occlusal surfaces. Different groups of tribological pairs were considered: tooth-tooth, tooth-restorative material (tooth-ceramic, tooth-resin-based-materials, and tooth-metal), and restorative-restorative materials. The lack of standardization of the wear tests impairs the direct comparison of the obtained results. However, it was possible to infer about the main wear mechanisms observed on the different classes of dental materials. Concerning ceramics, their toughness and surface finishing determines the wear of antagonist tooth. Abrasion revealed to be the main wear mechanisms at occlusal interface. In the case of resin-based composites, the cohesion of the organic matrix and the nature, shape, and amount of filler particles greatly influences the dental wear. The protruding and detachment of the filler particles are the main causes of abrasion of antagonist enamel. Metallic materials induce lower wear on antagonist enamel than the other classes of materials, because of their low hardness and high ductility. Most of the studies revealed plastic deformation and adhesive wear as the main wear mechanisms. Overall, more research in this area is needed for a better understanding of the mechanisms involved at the occlusal surfaces wear. This would be essential for the development of more suitable restoration materials.

## 1. Introduction

Teeth are extremely important and indispensable for phonetics, aesthetics, and mastication processes (incision, laceration, and grinding of food) [1,2,3]. Mastication involves the relative motion between teeth and/or food and can induce wear of the dental surfaces. From the tribological point of view, wear is defined as an undesirable alteration of a component’ dimension as a result of the gradual removal of discrete particles from the surfaces in contact and relative motion, caused mainly by mechanical actions. 

Teeth present an high wear resistance due to their compact and orderly microstructure of the external layer (enamel) and to the saliva action as lubricant and remineralization agent [2] (see Supplementary Materials for detailed information on teeth and saliva composition and function). However, an unavoidable cumulative tooth wear occurs due to mastication and to environmental/pathological factors which, depending on its extent, can be irreversible [3,4]. Mastication is the first step of digestion and has the main function of crushing and mixing foods, producing the bolus. The wear induced during mastication is influenced by foods characteristics, such as the rheology (flow behavior and deformation under shear, compression, and elongational fields), mechanical properties (hardness, brittleness), and geometrical properties (size and shape) [5,6]. Besides, foods and beverages ingestion leads to temperature (0–55 °C) and pH (1–10) variations, which may impose thermal stresses and demineralization to the dental tissues [7,8]. During mastication, typical teeth sliding distance are about 0.9–1.2 mm and normal forces range from 3 to 36 N [9]. These forces vary with the type of teeth and can reach much higher values (from 150–800 N [10]). Oral pathologies, such as bruxism, which corresponds to teeth grinding or clenching during sleeping, present a prevalence in the general population that ranges from 8%–31.4% [11]. It involves direct contact between teeth and therefore, can result in excessive wear [12,13]. Average forces between 105–420 N [14,15] and maximum forces of 900 N [16] were reported in literature. 

In tribology, the main wear mechanisms are abrasion (two or three-body abrasion), adhesion, fatigue, and tribochemical wear (chemical dissolution during wear) [17]. In dentistry, the term wear is wider and refers to the loss of dental tissue, being classified as attrition (wear at contact sites), abrasion (wear at non-contact sites), erosion (loss of material attributed to chemical effects), and abfraction (loss of dental tissue due to a fatigue process in the cervical part of the tooth) [18,19,20,21]. Understanding the wear mechanisms that occur on dental natural/artificial surfaces is essential to develop new approaches which allow to minimize the material loss and therefore guarantee the clinical lifetime, aesthetics, and adequate integration in the oral arcade [4,22,23]. This is becoming a real issue since life expectancy is increasing and teeth are needed to last longer [12].

Prosthetic dental materials should be able to stand the severe mechanical, chemical, and thermal oral requirements. They should also bond permanently to the tooth structure and exhibit properties similar to those of tooth tissues. Apart from wear, also traumas and tooth lesions such as caries, partial or overall tooth tissue loss may occur and consequently impair teeth health, having strong implications in the wellbeing and general health of the individuals [2,24]. Therefore, the repair/replacement of the damaged/missing tooth is of utmost importance, which requires the development of reliable artificial dental materials taking into consideration its corrosion, mechanical, and tribological behavior, cost, availability, biocompatibility, and aesthetics [25]. 

**Figure 1:**
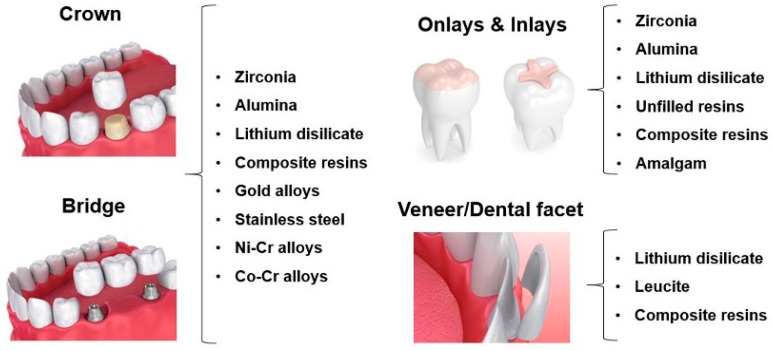
Materials currently used in dental restorations.

Nowadays dental restorations are produced using ceramics, resin-based materials, metals, and metal alloys [2,4,24]. Figure 1 presents an overview of the materials that can be used in dental restorations (crowns, bridges, onlays/inlays and veneers/dental facets—see Supplementary Materials for detailed information on prosthetic materials properties). However, till date, there is no material that can completely mimic natural human teeth regarding their biological, tribological, and mechanical properties. The wear of the occlusal surfaces may lead to disturbances in anatomy, aesthetics, phonetics, and mastication process due to the dental material loss and consequent lack of contact between the opposing surfaces [2,3,23,26,27].

This paper presents an overview of the works published in the last two decades, reporting wear studies on the interface of occlusal surfaces, following a tribological approach. The keywords used in the search were natural teeth, dental restorative materials, biotribology, wear, occlusal surfaces. Different groups of tribological pairs: tooth-tooth, tooth-restorative material (tooth-ceramic, tooth-resin-based materials, and tooth-metal) and restorative-restorative materials, were addressed. A comparative evaluation of the wear of the different materials groups was carried out in a final remarks section.

## 2. Wear at the Interface of Occlusal Surfaces

**Figure 2:**
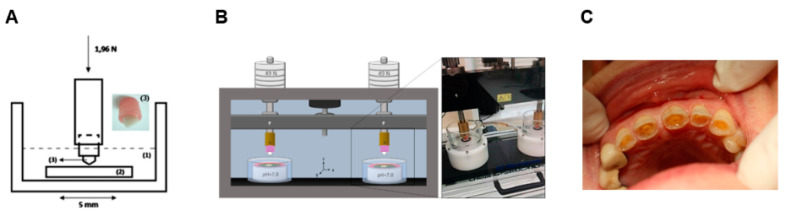
(**A**) Pin-on-plate configuration; (**B**) chewing simulator set-up; (**C**) clinical studies (Images (**A**) and (**B**) are reproduced from [28] and [29], respectively, with the permission of Elsevier, who is acknowledged).

Generally, wear studies involving dental materials are performed using simple configuration tests, such as pin-on-plate and pin-on-disc that allow a fast materials’ scoring and wear mechanisms understanding (Figure 2A). For the occlusal surfaces, chewing simulator tests can be used to better mimic the oral conditions (Figure 2B). Clinical studies are generally burdensome, time consuming, and involve ethical issues, but are crucial for the development of new materials and validation of testing methodologies/conditions (Figure 2C).

Most of the studies evaluate the wear resistance of dental materials using water or artificial saliva as lubricating media in two-body wear set-up (attrition, according to the dentistry nomenclature). However, there are few works that address the wear behavior of dental materials in the presence of particles in the interfacial media that mimic the role of food in three-body wear set-up (abrasion, according to the dentistry nomenclature). The scarce results show that the presence of these particles (e.g., poppy and millet seeds and poly(methyl methacrylate) during wear testing results in a reduction of the wear at the occlusal interface [30], since they are very soft and plastic, and in part are a shield that prevents the direct contact between the sliding surfaces. The selection/development of adequate restorative dental materials must take into consideration the wear induced both on the natural teeth and the restoration so as to minimize it [27,31].

### 2.1. Tooth-Tooth

Tooth wear is a complex process that depends not only on intrinsic factors (e.g., enamel characteristics such as thickness and hardness, tooth shape, position of the teeth in the maxillary, masticatory patterns) but also on extrinsic factors (e.g., food and beverages characteristics, oral hygiene habits). Pathological conditions (e.g., bruxism, xerostomia, gastroesophageal reflux disease (GERD)) can also contribute significantly to teeth wear [32]. 

There are a few studies regarding the enamel wear caused by opposing enamel (in vivo studies—see Table 1). Lambrechts et al. [33], who quantified the in vivo wear of human enamel, found that the vertical wear was 20 and 40 µm/year in premolar and molar regions, respectively. Similar values were observed by Mundhe et al. [34]. 

In terms of in vitro studies, it is difficult to compare the enamel wear values, since the works available (see Table 2) report them in different forms (vertical loss, worn area, and volume loss) and the wear tests were performed using different set-ups and operational conditions. Additionally, some authors underline the highest scattering of the obtained values, compared with other tribological pairs involving prosthetic materials. They attribute it to the high heterogeneity of human natural enamel, which is due to enamel composition and thickness variations related with the type of teeth, individual age, habits, diseases, medicine intake, and other factors [35]. Concerning to enamel wear mechanisms, abrasion, fatigue, and consequent delamination have been extensively observed in enamel-enamel tests. Arsecularatne et al. [36] proposed a microcracking mechanism that occurs under nominal elastic contact, similar to the one observed in ceramic materials. TEM analysis revealed two crack propagation mechanisms [37]: when the applied load is low, crack nucleation occurs at the enamel inter-rod discontinuities and propagates in the organic matrix; contrarily when the applied load is high, the crack propagates through the apatite crystals (transgranular fracture). The parallel propagation of the crack to the surface and then its inflection toward it, leads to the formation of lamellar wear particles (fatigue wear). The roughening of the surface due to the delamination of enamel and the released wear particles leads to two and three-body abrasion, which increases the enamel wear rate [36]. A compact layer (tribolayer) may be formed on the enamel surface due to the aggregation and compaction of the wear particles released from the tooth surface during the wear tests [37,38], which acts as a protective layer against enamel wear. According to Zheng et al. [38], the presence of a tribolayer leads to an increase of the real contact area and consequently to the decrease of the contact stress, resulting in a lower enamel wear. 

**Figure 3:**
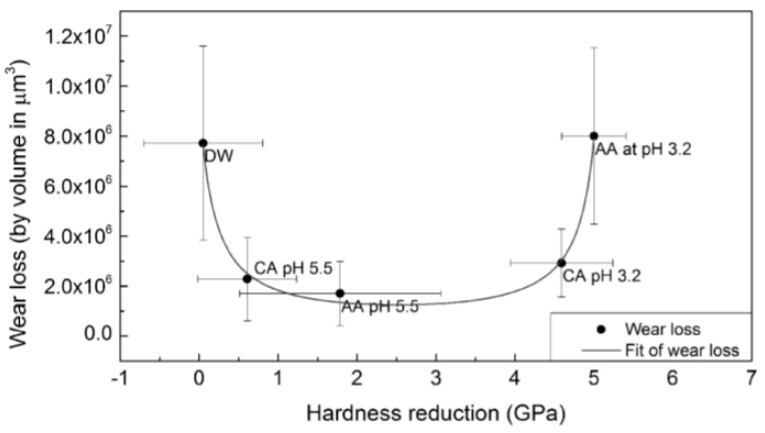
Enamel wear loss as a function of hardness reduction. DW: distilled water (pH = 7); CA: citric acid; AA: acetic acid. (reproduced from [41]).

The influence of the presence of acids on enamel wear during testing was evaluated by some authors. The results are not consensual, which may be related to the soften action of the acids on enamel surface and the testing conditions. Some studies showed that wear tests conducted under acidic conditions lead to a higher enamel wear than tests carried out in neutral solutions [39]. Contrarily, others found lower enamel wear when tested in the presence of acidic media [40,41,42,43,44]. In fact, Wu et al. [41] found that the influence of acids on enamel wear depends on reduction of hardness that these induce on the enamel surface. According to this work, the wear loss decreases significantly for hardness reductions of the order of ≈0.5 GPa and is kept approximately constant until a threshold of hardness reduction of ≈4.5 GPa. Above this value the wear loss increases again, reaching values similar to those found in the absence of acids (Figure 3). The authors explain that the removal of material is done through the shaving of the softened enamel layer (produced by the attack of the acid). If the reduction of enamel hardness does not overcome the threshold, the shaving wear mechanism results in lower enamel loss than that induced by microfatigue. This occurs for neutral solutions and is responsible for the formation of lamellar particles (delamination) that lead to considerable material loss.

### 2.2. Tooth-Ceramic

Ceramic materials generally induce a higher wear on the antagonist natural teeth than the other classes of restorative materials do. There are several studies focusing the wear suffered by the natural tooth against ceramic restorations and also the wear mechanisms that occur between these occlusal surfaces. Mundhe et al. [34] measured and compared the in vivo wear of enamel opposing zirconia and PFM (porcelain fused to metal) ceramic crowns after 1 year. Similarly, to the enamel-enamel studies referred in the previous section, they observed a higher wear in the molar teeth, compared to pre-molars. Besides, they concluded that PFM ceramic crowns induced a vertical wear on the enamel antagonist 1.4–1.6 times higher than zirconia and 4–5.1 times higher than enamel. In addition, Suputtamongkol et al. [45] investigated the clinical performance and wear characteristics of lithium-disilicate based ceramic crowns when tested against human enamel. They found that after 1 year, the mean occlusal wear volumes for premolar ceramic crowns and their antagonists were similar, while for molars, the natural enamel suffered a higher wear. Aladağ et al. [46] evaluated the in vivo wear of lithium disilicate (IPS E-max CAD, EM) and zirconia-reinforced lithium silicate (Vita Suprinity CAD, VS) against dental enamel for 6 months. They observed that both materials induced similar enamel wear, being the values of the same order as those observed by Suputtamongkol et al. when normalized for the same test period. On the other hand, EM suffered higher wear than VS did. 

In vitro studies (Table 2) showed that even though zirconia presents a high hardness, it usually leads to a lower enamel wear compared to glass-based ceramics (lithium disilicate, leucite, feldspar) and glaze/veneers applied to ceramic restorations [51,62,65,67]. In fact, the work published in the literature demonstrates that the predominant dental wear causes are the prosthetic materials roughness/surface finishing, microstructure, fracture toughness, and environmental factors [21,53,68,69,70].

**Figure 4:**
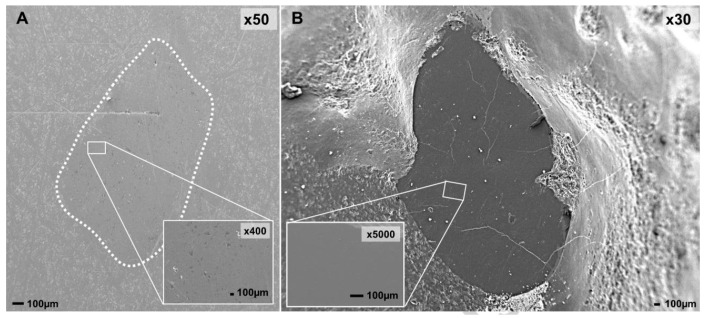
SEM images of zirconia (**A**) tested against natural human dental cusps (**B**) in a chewing simulator (reproduced from [28]).

Santos et al. [28] compared the wear performance of some ceramic dental materials (zirconia, leucite and zirconia veneered) when tested against natural teeth and concluded that zirconia led to the lowest wear on both occlusal surfaces (Figure 4A,B). On the other hand, leucite and zirconia Veneered induced the highest teeth wear. Regarding the wear mechanisms between the occlusal surfaces, polishing wear was observed for the zirconia/tooth pair. Contrarily, microfracture-based wear mechanisms associated with abrasive wear was observed for the other tribological pairs. 

**Figure 5:**
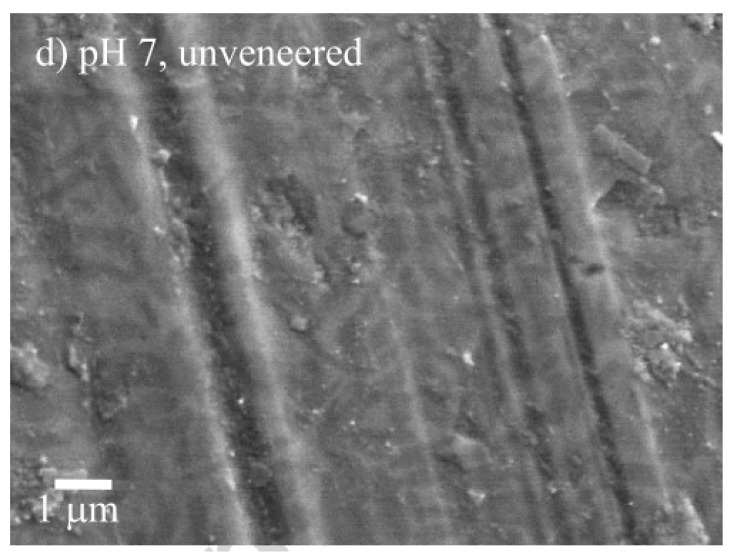
Wear tracks in lithium disilicate plates after pin-on-plate tests in artificial saliva at pH = 7 using natural cusps as pins (reproduced from [50]).

In a study conducted by Wang et al. [9], the wear behavior of four dental ceramics (polished and rough zirconia, hot-forged lithium disilicate glass ceramics and silicates-based veneer porcelain) was accessed. It was observed that the sliding of the worn surfaces of enamel against rough zirconia, lithium disilicate glass ceramics and veneer porcelain led to furrows and granular debris, indicating abrasive wear. On the other hand, chipping flake and pit-like structure after stripping and crack, which indicates fatigue wear, was observed on the enamel surface while sliding against polished zirconia. The authors concluded that the wear performance of zirconia can be improved with the use of adequate polished zirconia surfaces. Also, Ghazal et al. [71], who performed a study with natural teeth against zirconia with different finishing degree, observed that the dental wear increased with zirconia roughness. Kim et al. [64] performed wear tests using three different zirconia dental ceramics (Prettau, Lava, Rainbow), lithium disilicate, and porcelain against dental enamel and observed that lithium disilicate and porcelain induced higher wear on enamel and on themselves than the three types of zirconia did. Bolaca et al. [52] studied the wear of primary tooth enamel against monolithic zirconia and lithium disilicate. Lithium disilicate induced the highest wear on enamel because of its lower fracture toughness. This led to the surface chipping/fracture during the wear test, resulting in the formation of sharp edges and broken glass particles that may have increased the amount of antagonist tooth wear. Choi et al. [54] compared the wear induced on primary teeth by yttria stabilized zirconia, lithium disilicate, and leucite and also quantified the amount of wear for each prosthetic material. They concluded that leucite caused the highest amount of wear on primary teeth followed by lithium disilicate. Again, this is explained by the low fracture toughness, but also by the low flexural strength of leucite and lithium disilicate comparatively to zirconia. For leucite and lithium disilicate, the enamel wear is caused by the formation of glass-ceramic chips/debris that function as abrasive particles inducing a three-body wear mechanism [72]. The size and shape of these particles influence enamel wear [73]. Zirconia led to the lowest wear on the antagonist primary teeth due to its high hardness, flexural strength, density, and fracture toughness that prevents the formation of surface microfractures. Regarding the prosthetic materials’ wear, lithium disilicate and leucite showed the highest amount of wear: glass ceramics are sensitive to fatigue, and thus, the wear process initiates with the formation of cracks on the materials’ surface. These cracks are propagated by repetitive loading, causing an eventual material loss [74]. In another work, Figueiredo-Pina et al. [50] proposed an explanation for the wear mechanism that occurs in the pair enamel/lithium disilicate as follows: the sliding of enamel against the lithium disilicate restoration led to microcracking around the nanometric hydroxyapatite (HA) prismatic crystals, resulting in dental wear debris formation (Figure 5). Some HA particles remain attached to the dental surface and the glass matrix of the prosthetic material fractures due to stress concentration. Because of the localized high contact stress, there is the cracking of the glass matrix of the prosthetic material, releasing three-body particles. These particles induce ploughing and cutting on the both contacting surfaces. A tribolayer is formed mainly on the enamel surface, by agglomeration and compaction of the wear debris from the two opposing surfaces. This was not observed in a posterior study, from the same research group, where, instead of a pin-on-plate apparatus, a chewing simulator was used [28]. In fact, in this case, the wear particles remain less time between the contacting surfaces, reducing the possibility of formation of such compact protective tribolayer on the dental surface. A pin-on-plate study showed that the extension of dental particles agglomeration on the enamel surface and delamination is dramatically reduced when a food slurry is present in the interfacial medium, leading to a reduction of wear of both contacting surfaces [66]. 

Concerning the influence of the acids on the enamel-ceramic pair during testing, it has not been subject of much attention. Acids can attack the enamel as well as some ceramic materials. Figueiredo-Pina et al. [50] showed that veneered and unveneered lithium disilicate induced higher enamel wear for tests carried out in distilled water at pH = 7 than in citric acid at pH = 3. Smoother enamel surfaces were obtained at pH = 3, which is in agreement with the work of Wu et al. referred above [41]. Contrarily, Ratledge et al. [39] observed that the wear of enamel against Vitadur-N glazed porcelain and unglazed IPS Empress ceramic was higher for tests carried out in citric acid (pH = 4) than in water. The difference in the observed behaviors may be related with the testing conditions, namely the applied load, that is much higher in the study of Ratledge et al. [39].

Bacteria can also change the tribological response of enamel against ceramics. Figueiredo-Pina et al. [75] carried out tribological tests using the pair enamel-zirconia in the presence of saliva and a non-cariogenic *Streptococcus salivarius* biofilm. They observed that the presence of the *S. Salivarius* biofilm changes the enamel-zirconia pair triboactivity, decreasing dental wear. Regarding wear mechanisms, the worn cusp surface shows less delamination features and a lower tribolayer thickness. According to the authors, more research is needed to fully understand the influence of the biofilms on enamel wear. 

**Figure 6:**
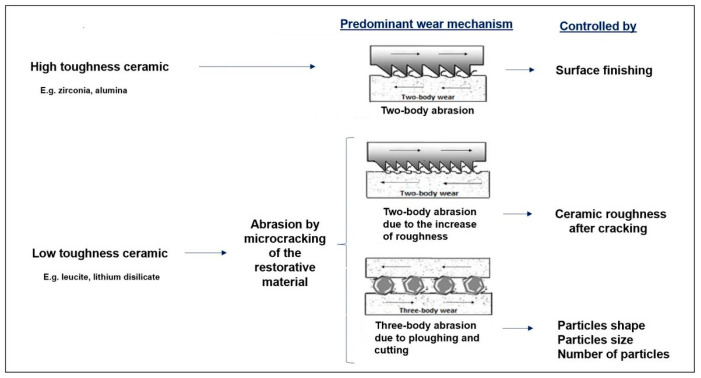
Scheme of the wear mechanisms occurring in the interface of enamel/ceramic prosthetic material.

In resume, ceramics mainly present wear mechanisms associated with two-body and three-body abrasion, which depend on the ceramic toughness (Figure 6). For high toughness ceramics (e.g., zirconia), the prosthetic material wear is neglectable and the dental wear results from the penetration of the harder ceramic surface asperities that cut/plough the softer enamel surface (two-body abrasion). Thus, the wear is controlled by the prosthetic material surface finishing. For low toughness ceramics (e.g., leucite, lithium disilicate), the contact stress produced during mastication leads to abrasion by microcracking of the prosthetic material, increasing its roughness and releasing wear debris from its surface (three-body particles). The hard-rougher surface (which induces two-body abrasion) associated with the presence of hard three body particles (responsible by three-body abrasion) leads to a dramatic increase of the dental wear. 

### 2.3. Tooth-Resin-Based Material

The increasing use of resin-based restorative materials, mainly of composite resins, on the occlusal surfaces has brought some attention to the wear suffered by the opposing dental enamel, since these materials can induce its abrasion in different extents. According to some authors, natural teeth are abraded because of the high wear resistance of the composite resins and the high surface roughness due to the presence of coarse filler particles [76,77,78]. The presence of a finer particle size (1 µm or less) in high concentration within the polymer matrix of the composite results in less interparticle spacing, more protection of the softer resin matrix, and less filler plucking, all contributing to a high wear resistance of the material [79,80]. The filler particles induce enamel wear by protruding from the abraded resin matrix, being the amount of enamel wear directly correlated with the hardness of the composite resin [35]. The size, shape, hardness, and content of the filler particles of a composite resin are determinant for the wear caused on the antagonist teeth [78]. 

Kramër et al. [48] carried out a clinical study to evaluate the wear at the interface between resin composite restorations and tooth over eight years. They used a fine hybrid resin composite, Tetric Ceram, and a nanohybrid composite resin Grandio. The results showed that the wear of enamel was always superior to that observed for the both resins. Moreover, Grandio suffered higher wear over time and induced a superior height loss in the teeth. It is underlined that a decrease on the vertical height loss was verified during the study period, reaching less 44% and 49%, for resin and enamel, respectively, in the eighth year comparatively to the second year. In another study [81], the same group found that both resins significantly changed over time for all criteria evaluated. However, they still showed an adequate clinical performance after 8 years of service. Concerning the teeth, their integrity was significantly affected, enamel cracks and chippings being observed over time. In a 3-year randomized clinical trial, Palaniappan et al. [82] compared the clinical performance of posterior composite restorations carried out with an hybrid resin (Tetric Ceram), a microfilled hybrid resin (Gradia Direct Posterior), and a nanohybrid resin (Tetric EvoCeram, TEC), against human enamel. Fatigue crack propagation was observed through SEM, for the three composites, which suggests that none of the restorations could sustain the fatigue induced by the cyclic stress during mastication. The nanohybrid and the microhybrid resin restorations were less susceptible to pitting over time, due to their smaller filler particles. Regarding wear quantification, the composite resin that showed the highest wear resistance was the nanohybrid, while the one that presented the lowest was the microfilled hybrid. In another in vivo study, Palaniappan et al. [47] compared the performance of a nanocomposite (Filtek Supreme) and a microhybrid composite (Z100) opposing enamel after three years of clinical use and found that both enamel and composite resins’ wear was higher for Z100. Also, it was observed that the volume loss of both composite resins was significantly higher than that found on enamel. The same authors [49] evaluated the five-year clinical wear performances clinical of the same type of composite resins (Filtek Supreme and Z100) applied in restorations and arrived at similar conclusions. 

**Figure 7:**
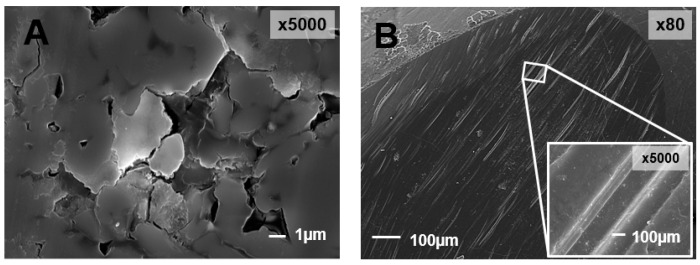
SEM images of (**A**) Vita Enamic after testing against natural human dental cusps in a chewing simulator, showing the degradation of the organic matrix of the prosthetic material; (**B**) surface of the dental cusp after the wear test against Vita Enamic, showing abrasive wear (reproduced from [28]).

Regarding in vitro studies, Sripetchdanond et al. [35] conducted wear tests with enamel against a composite resin and observed that the wear mechanisms present in both enamel and restorative material were polishing and abrasive wear, respectively. Moreover, they found that the mean vertical wear induced by the resin on enamel was about six times lower than that found on enamel-enamel system tested in the same conditions. Santos et al. [28] studied the wear mechanisms of the pair Vita Enamic^®^ (a polymer infiltrated ceramic (PIC))/enamel and observed that Vita Enamic^®^ suffered a high wear, because of the degradation of its organic binder phase, probably due to fatigue. Figure 7A shows the degradation of the organic phase matrix of Vita Enamic with the consequent release of the ceramic hard particles (three-body particles), which scratch the dental cusp (Figure 7B).

Gazhal et al. [60] studied the wear of enamel against two resins (a nano-filled composite resin (NCR) and an acrylic resin (AR)) and found that the nano-filled composite resin induced a lower wear on antagonist enamel than the acrylic resin did. This is explained by the composition of each resin: the NCR has inorganic fillers, which protect the resin matrix during the wear test, leading to a smoother surface than that of acrylic resin. Concerning the resins’ wear mechanisms, pull-out and fatigue wear were reported. Jang et al. [61] evaluated the wear behavior of three composite resins (Gradia Direct microhybrid, Filtek Z250 microhybrid and Filtek Z350 nanocomposite) against human enamel and found that the mean enamel vertical loss was lower against Gradia Direct microhybrid composite resin. Regarding the wear mechanisms observed for the three resins, Gradia Direct microhybrid revealed microcracking (low extent) and scratching; Filtek Z250 microhybrid showed microcracking between the filler and the matrix, with fillers’ pull-out, and finally, for Filtek Z350 nanocomposite, an intensive plastic deformation with accumulation of resin particles on the surface was observed. Condon et al. [83] used a multi-mode oral simulator to evaluate the wear of enamel against composite resins and observed that the antagonist enamel wear was higher for the composites containing the largest filler particle sizes.

Besides the size, also the nature and amount of the filler affects the wear behavior. Suzuki et al. [63] measured the wear induced by ten commercially available composite resins on enamel and concluded that composite resins containing zirconium silicate or quartz fillers caused higher enamel wear than composite resins containing microfilled or barium silicate-fillers. This increased in wear may be attributed to the harder filler particles. In another study, Suzuki et al. [84] evaluated the wear characteristics of seven composite restorative resins against human enamel and observed that the composite with the highest filler loading (92 wt% of lanthanum oxide) led to the highest enamel wear.

Concerning the effect of acids on composite resins, Correr et al. [85] studied the influence of the exposure to citric during wear tests in a chewing simulator using several composite resins against dental enamel and found that for all the tested resins, the wear resistance is lower than that found in neutral conditions. However, the antagonist enamel tested in acidic medium only revealed a slight reduction in the wear, sometimes neglectable. In addition, the enamel cusps tested in acidic medium showed a more polished surface. 

Overall, resin-based composite materials induce wear on the antagonist tooth surface, whose extent depends on the characteristics of the polymeric matrix and on the hardness, concentration, size, and shape of the filler particles. For composite resins with microfillers, the adhesion of the filler particles to the polymeric matrix determines the wear suffered by the antagonist teeth and also by the composite resin itself. If this adhesion is weak, the filler particles will be easily released from the matrix. The loss of the mechanical support of the filler particles can also lead to its fracture, releasing particles with sharper edges, which in turn increase the three-body abrasion [28]. If this adhesion is strong, while the matrix is being abraded during wear, the filler particles begin protruding through the matrix, increasing roughness, and may lead to two-body abrasion. The results show that the harder the filler particles, more severe is the wear. For nanofilled composite resins, because of the low size of the fillers, the wear is not so sensitive to the fillers’ hardness. In this case, the wear mechanisms are mainly related to the plastic deformation of the composite resin, leading to lower enamel and composite wear and to more polished worn surfaces. 

### 2.4. Tooth-to-Metal

Nowadays, mostly in occidental countries, metal is not the chosen material to be used in dental crowns mainly because of aesthetic reasons. Metal’s hardness plays an import role in the wear induced on the antagonist teeth and on itself. According to Fisher et al. [86], “For most materials, metal in particular, the wear resistance is believed to be directly proportional to the hardness.” Some metals such as stainless-steels crowns are usually the first choice to repair defects in primary teeth caused by caries. Although this is considered an effective and efficient method of tooth restoration in pediatric dentistry, the aesthetic concerns seem to be a critical issue. Choi et al. [54] conducted a study on the wear of the pair enamel/stainless steel and found that the occlusal forces were absorbed by the ductility of the steel. Also, through SEM images, it was observed that enamel surface presents abrasion and stainless-steel shows plastic deformation and abrasion by ploughing. Pereira et al. [59] investigated the wear of stainless-steel opposing enamel and observed that there was some adhesion of dental particles to the surface of stainless-steel. Ratledge et al. [39] investigated the wear produced by amalgam and observed that adhesive wear was the main wear mechanism, since some material transfer from amalgam was detected on the enamel surface. Wang et al. [9] investigated the wear behavior of enamel against gold-palladium and nickel-chromium alloys and concluded that both alloys induce similar enamel wear. Also, they observed that the wear mechanisms present on enamel were adhesive wear induced by the Au-Pd alloy and fatigue and adhesive wear induce by Ni-Cr alloy. Lee et al. [55] observed the wear mechanisms on the surfaces of the pair enamel/type III gold. SEM images revealed adhesive wear on the enamel surface since there was some gold transfer to enamel, and a polished surface of gold. Finally, Ramp et al. [56] and Suzuki et al. [63] measured the wear of enamel when opposed to gold and found similar values (0.019 mm^2^ and 0.016 mm^2^, respectively).

In resume, the metals and their alloys present similar or lower hardness than enamel, which leads to wear mechanisms associated to plastic deformation on its surface and material transference (adhesive wear). 

### 2.5. Restorative-to-Restorative Materials

Apart from the wear suffered at the occlusal interface of enamel-restorative materials, wear may also occur at the interface of restorative-restorative materials. However, no clinical studies were found concerning this topic. In turn, there are several in vitro studies in the literature that address the wear performance of different restorative pairs and therefore can be used to foresee the tribological behavior of the involved materials when used in the occlusal interface. Although in the literature, the tribological tests are usually carried out against zirconia counterbodies, also alumina, stainless steel and Co-Cr alloys are used (Table 3). D’Arcangelo et al. [87,88] conducted chewing simulator studies to evaluate the tribological behavior of several restorative materials (type III gold alloy, lithium disilicate, feldspathic porcelain and different composite resins) against zirconia. They observed that type III gold alloy presented the lowest wear among all materials. Lithium disilicate, feldspathic porcelain, and microhybrid resin composite showed similar wear. The nanocomposite resins (Enamel plus HRi, Filtek Supreme XTE, and Ceram.X duo) presented the highest wear. In another work, the same group [89] studied the two-body wear resistance of different materials against antagonist cusps made of the same material and found that monolithic zirconia led to the lowest wear values. Borrero-Lopez et al. [90] performed a pin-on-plate wear study where zirconia, lithium disilicate, feldspathic ceramic, and two types of ceramic–polymer composites were tested against zirconia. They observed that zirconia and one of the ceramic–polymer composite Enamic presented lower wear compared to the other materials. Lithium disilicate and the ceramic–polymer composite Lava Ultimate suffered higher wear, showing signs of abrasion. In addition, microcracking was observed for lithium disilicate and pull-out and fatigue for Lava Ultimate. In another study, Esquivel et al. [91] compared the wear of three unfiled resins and one nano-hybrid composite resin against zirconia and observed that the nano-hybrid composite worn less comparatively to the other resins, which allowed concluding that the filler content is responsible for the lowest wear of the resin. Kootathape et al. [92] evaluated the wear of several composite resins (Durafill VS (DUR), Clearfil AP-X (APX), Filtek Z250 (Z250), Filtek Supreme XT (FIL), Kalore (KAL), MI Flow (MFL), Venus Diamond (VED), and Venus Pearl (VEP)) against zirconia in three lubricating media (water, poppy slurry, and PMMA slurry) and found that the wear and morphology of each worn surface is determined by the type of composite and media used. DUR, KAL, and MFL presented high wear resistance in water. In the presence of PMMA slurry, DUR, Z250, and FIL showed moderate wear, while APX, KAL, and MFL showed higher wear. In the interfacial medium with poppy seed slurry, DUR was the composite resin that presented the highest wear. Through SEM analysis, it was observed that Z250, FIL, and MFL suffered abrasive wear in water. It was also observed that KAL and MFL in the interfacial media containing poppy seed slurry were heavily destroyed, while VED and VEP appeared very smooth. In the presence of PMMA slurry, KAL and MFL suffered abrasion and their surface presented many cracks. Contrarily, VEP did not present cracks and its surface remained smooth. Ghazal et al. [93] compared the wear of a nanofilled composite resin and of a feldspathic ceramic against zirconia and alumina and observed that both counterbodies induced a lower wear on the nanofilled composite resin. In addition, each material presented the same wear mechanisms against zirconia and alumina: the nanofilled composite resin presented abrasion and delamination and the feldspathic ceramic presented abrasion and microcracking, the latter mechanism being responsible for the higher wear of the material. Silva et al. [94] used a counterbody of alumina to study the wear of zirconium-lithium silicate (ZLS) glass-ceramic and of a polymer-infiltrated ceramic network and observed that ZLS presented the highest wear resistance, because of the absence of a debris’ layer on its surface during sliding. Yilmaz et al. [95] evaluated the wear performance of a nanofilled, a microfilled, and a nanohybrid composite resin against alumina and observed that the microfilled composite resin presented the lowest wear. In another study [64], zirconia, lithium disilicate, and porcelain were tested against a feldspathic porcelain in a chewing simulator and the highest wear was observed for lithium disilicate. The other tested materials presented similar wear. Barkmeier et al. [96] determined the wear induced on different composite resins by stainless steel and found that the microhybrid resin Z100 showed a lower wear than the resins microhybrid Filtek Z250, nanohybrid Tetric EvoCeram, nanocomposite Filtek Supreme Plus and nanohybrid Esthet X, the latter being the one presenting the highest wear. Alarcon et al. [97] studied the wear of a microhybrid composite (Filtek Z250), type III gold alloy, and porcelain against Co-Cr alloy and observed that porcelain showed the highest wear resistance, presenting neglectable wear. Yap et al. [98] used a metallic counterbody (stainless steel) against several composite resins to study the influence of the water absorption by the resins on their wear behavior and concluded that it impaired their tribological performance.

Overall, the high number of materials combinations on restorative-restorative tribological systems hinders a straight comparison between them. However, concerning metals, it can be stated that gold tested against zirconia suffers a lower wear than composite resins or ceramic materials. Also, one can conclude that zirconia induces lower wear on zirconia counterbodies than on low toughness ceramics. Finally, a global analysis shows that abrasion is the most common wear mechanism for ceramics and composite resins when tested against zirconia and alumina surfaces.

### 2.6. Final Remarks

Because of the lack of standardization, it is difficult to compare the results of the wear tests among the different studies. In vivo tests that allow to validate the in vitro findings are scarce. An effort has been done to approach in vivo conditions, e.g., performing chewing simulation studies. However, there are several issues that limit the mimetization of the real conditions in mastication. For example, the jaw kinematics is not fully recreated, as well as the sequence of applied loads. On the other hand, lubrication is generally carried out with water, saline solutions, or artificial saliva, neglecting the effect of the organic compounds present in natural saliva that are responsible for the formation of a salivary pellicle on the enamel surface. Such pellicle plays a key role in the teeth protection against chemical attack and may also strongly affect the wear. As far as the authors know, no studies exist on the later topic. Another aspect that has been barely addressed is the influence of microorganisms present in the oral cavity, both dispersed in the saliva and adsorbed to dental material surfaces forming a dental plaque. Biofilms viscoelastic properties and changes induced in occlusal materials by the action of microorganisms’ metabolic products may change the tribological systems behavior. Another aspect that usually is not considered in the wear studies is the effect of the presence of food. Most of the works do not introduce any three-body particles during the wear tests. Few use particles that are far from simulating the diary human diet.

The analysis of the collected research data shows that in general, metals and their alloys worn less and induce the lowest enamel wear comparatively to other restorative materials. In fact, there are several comparative studies that report that gold and their alloys [56,57,58], stainless steel [54], Ni-Cr alloys [9] and amalgam [39] present better tribological performance than ceramics and composite resins. This is explained by the low hardness and high plastic deformation generally exhibited by these materials. Adhesive wear is commonly observed on the antagonist enamel surface and also on the metals’ surface. Concerning ceramics, the wear behavior is determined by their toughness. For the toughest materials, such as zirconia, the wear is neglectable. It has also been reported that this material leads to lower enamel wear values [52,61] than less tough ceramics like porcelains, lithium disilicate and leucite [52,60,61]. Composite resins present intermedium wear values between the two types of ceramics. Both in low toughness ceramics and composite resins, the wear mechanisms involve the release of ceramic particles and roughening of the surface. These particles (three-body) and the consequent increase of roughness surface (two-body) enhance abrasion, leading to an increase of enamel wear. The differences may be due to the amount, size, shape, angulation, and hardness of the detached particles and the roughness and hardness of the worn surface. More studies are needed in order to clarify this topic.

Concerning the restorative-restorative materials wear, the analyzed studies showed that zirconia is one of the most tested materials and is among the ones that suffer lower wear. For vitroceramics and resins the results’ trend is not well-defined, since these materials present different characteristics resultant from their specific compositions and different manufacturing methods.

## 3. Conclusions

In the present study, a review of the literature on the wear mechanisms at dental occlusal surfaces (tooth-tooth, tooth-restorative material and restorative-restorative material) was performed. The different classes of restorative materials (ceramics, resin-based composites, and metals) were addressed.

When the tribological systems involve ceramics, the fracture toughness and surface finishing constitute the main variables that determine the wear of the opponent enamel surfaces. Abrasion is the main wear mechanism. Usually, tougher ceramics induce lower enamel wear, because they reduce the formation of hard three-body particles.

With resin-based composites, the protruding and detachment of the ceramic filler particles from the organic matrix are the main issue, since they are responsible for the wear on the antagonist tooth surface through two-body and three-body abrasion, respectively. The damage extent depends on the nature, shape, and concentration of the filler particles.

Finally, with metallic materials, adhesion is the main wear mechanism. This class of materials leads to the lowest dental wear.

Although research in the dental biotribology field is currently ongoing, there is still a long way to fully understand the wear mechanisms occurring on the restorative and natural dental materials. This is crucial for the development of new best performing materials for dental restorations.

## Figures and Tables

**Table 1:** Tribological in vivo studies concerning the wear at the occlusal enamel-restorative material interface.

Ref.	Tribological Pair	Enamel Wear	Restorative Material Wear
[33]	enamel/enamel	Premolars: 20 µm/year Molars: 40 µm/year	-
[34]	PFM ceramic/enamel	Premolars: 69.20 ± 4.10 µm/year Molars: 179.70 ± 8.09 µm/enamel	-
	zirconia/enamel	Premolars: 42.10 ± 4.30 µm/year Molars: 127.00 ± 5.03 µm/enamel	-
	enamel/enamel	Premolars: 17.30 ± 1.88 µm/year Molars: 35.10 ± 2.60 µm/enamel	-
[45]	lithium disilicate/enamel	Premolars: 0.21 ± 0.06 mm^3^/year Molars: 0.50 ± 0.22 mm^3^/year	Premolars: 0.19 ± 0.065 mm^3^/year Molars: 0.34 ± 0.08 mm^3^/year
[46]	lithium disilicate/enamel	0.26 ± 0.17 mm^3^/6 months	0.27 ± 0.16 mm^3^/6 months
	zirconia reinforced lithium silicate/enamel	0.28 ± 0.14 mm^3^/6 months	0.14 ± 0.14 mm^3^/6 months
[47]	microhybrid composite resin (Z100)/enamel	0.2 ± 0.1 mm^3^/3 years	0.4 ± 0.2 mm^3^/3 years
	nanocomposite resin (Filtek Supreme)/enamel	0.1 ± 0.1 mm^3^/3 years	0.3 ± 0.1 mm^3^/3 years
[48]	nanohybrid composite resin (Grandio)/enamel	135 ± 104 µm	108 ± 88 µm
	fine hybrid resin composite (Tetric Ceram) /enamel	110 ± 58 µm	98 ± 53 µm
[49]	nanofilled restoration (Filtek Supreme)/enamel	0.31 ± 0.1 mm^3^/5 years	0.82 ± 0.2 mm^3^/5 years
	microhybrid restoration (Z100)	0.47 ± 0.2 mm^3^/5 years	1.04 ± 0.9 mm^3^/5 years

**Table 2:** Tribological in vitro studies concerning the wear at the occlusal enamel-restorative material interface.

Ref.	Type of Test	Operational Conditions	Lubricant	Tribological Pair	Enamel Wear	Restorative Material Wear	Wear Mechanisms and Features	
							Enamel	Restorative Material
[35]	Pin-on-disc	25 N 4800 cycles 20 rpm	Distilled water	enamel/enamel	8.81 ± 5.16 µm	-	Delamination	Delamination
				enamel/zirconia	1.83 ± 0.75 µm	-	Delamination	Few scratches
				enamel/lithium disilicate	7.32 ± 2.06 µm	-	Abrasion	Massive fracture
				enamel/composite resin	1.37 ± 0.81 µm	-	Polishing	Abrasion (?)
[50]	Pin-on-plate	1.96 N 21,600 cycles 1 Hz Stroke: 3 mm	Artificial saliva (pH = 3 & 7)	unveneered lithium disilicate/enamel (pH = 3)	1.3 × 10^−^^3^ mm^3^/Nm	0.2 × 10^−^^3^ mm^3^/Nm	Abrasion/ polishing	Microfracture
				unveneered lithium disilicate/enamel (pH = 7)	2.5 × 10^−^^3^ mm^3^/Nm	0.1 × 10^−^^3^ mm^3^/Nm	Abrasion and tribolayer formation	Microfracture and dental transfer
				veneered lithium disilicate/enamel (pH = 3)	1.75 × 10^−^^3^ mm^3^/Nm	0.3 × 10^−^^3^ mm^3^/Nm	Abrasion/ polishing	Microfracture
				veneered lithium disilicate/enamel (ph = 7)	2.9 × 10^−3^ mm^3^/Nm	0.5 × 10^−^^3^ mm^3^/Nm	Abrasion and tribolayer formation	Microfracture and dental transfer
[39]	Cycling machine	40 N 25,000 cycles	A: Citric acid (pH = 4) W: control group	enamel/enamel	W:0.6 ± 0.4 mm^2^	-	Chipping	Chipping
					A: 1.2 ± 0.6 mm^2^			
				enamel/amalgam	W:0.3 ± 0.3 mm^2^	-	-	Dental transfer
					A: 0.5 ± 0.4 mm^2^			
				enamel/conventional composite	W:0.7 ± 0.5 mm^2^	-	-	Scratching and pull-out (?)
					A: 1.2 ± 0.7 mm^2^			
				enamel/microfilled composite	W:0.4 ± 0.4 mm^2^	-	-	Scratching (?)
					A: 0.5 ± 0.6 mm^2^			
				enamel/glazed porcelain	W:1.2 ± 0.6 mm^2^	-	-	Abrasion (?)
					A: 1.5 ± 0.4 mm^2^			
				enamel/unglazed metal-free ceramic	W:0.8 ± 0.6 mm^2^	-	-	Abrasion (?)
					A: 1.3 ± 0.9 mm^2^			
[28]	Chewing simulator	49 N 3600 cycles 1 Hz Stroke: 0.7 mm	Artificial saliva (pH = 7)	Vita Enamic^®^/enamel	0.09 ± 0.01 mm^3^	0.24 ± 0.04 mm^3^	Abrasion, delamination	Abrasive wear and pull-out
				zirconia/enamel	0.08 ± 0.01 mm^3^	0	Polishing wear	No abrasion
				leucite/enamel	0.19 ± 0.01 mm^3^	0.14 ± 0.02 mm^3^	Abrasion	Microfracture
				zirconia veneered/enamel	0.21 ± 0.02 mm^3^	0.19 ± 0.09 mm^3^	Abrasion	Microfracture
[51]	Chewing simulator	5 kg 240,000 cycles 0.8 Hz Stroke: 0.3 mm	Water (5 °C/55 °C)	polished feldspathic porcelain/enamel	0.119 ± 0.059 mm^3^	-	-	-
				polished zirconia/enamel	0.031 ± 0.033 mm^3^	-	-	-
				polished glazed zirconia/enamel	0.078 ± 0.063 mm^3^	-	-	-
[9]	Chewing simulator	4 N 5000 cycles 2 Hz Stroke: 1 mm	Artificial saliva	polished zirconia/enamel	200–300 µm	-	Fatigue and adhesive wear	-
				rough zirconia/enamel	200–300 µm	-	Abrasive and fatigue wear	-
				lithium disilicate/enamel	600 µm	-	Abrasive wear	-
				porcelain/enamel	500 µm	-	Abrasive wear	-
				Au-Pd/enamel	100 µm	-	Adhesive wear	-
				Ni-Cr/enamel	100 µm	-	Fatigue and adhesive wear	-
[52]	Chewing simulator	50 N 100,000 1.6 Hz Stroke: 2 mm	Artificial saliva (5 °C/55 °C)	zirconia/enamel	2.66 ± 0.65 mm^3^	-	-	-
				lithium disilicate/enamel	3.84 ± 0.7 mm^3^	-	-	-
				resin nanoceramic/ enamel	3.48 ± 0.71 mm^3^	-	-	-
				nanohybrid composite resin/enamel	3.68 ± 0.76 mm^3^	-	-	-
				primary tooth enamel/enamel	1.66 ± 0.42 mm^3^	-	-	-
[53]	Two-body wear testing device	75 N 100,000 cycles 1.2 Hz	Water	lithium disilicate glass/enamel	0.33 ± 0.12 mm^3^	0.10 ± 0.03 mm^3^	-	-
				leucite-reinforced glass/enamel	0.42 ± 0.09 mm^3^	0.11 ± 0.02 mm^3^	-	-
				zirconia/enamel	0.07 ± 0.03 mm^3^	0.23 × 10^-3^ ± 0.18 × 10^−^^3^ mm^3^	-	-
				feldspathic porcelain/enamel	0.62 ± 0.27 mm^3^	0.05 ± 0.03 mm^3^	-	-
				enamel/enamel	0.40 ± 0.16 mm^3^	0.08 ± 0.08 mm^3^	-	-
[54]	Chewing simulator	50 N 100,000 cycles 0.8 Hz Stroke: 2 mm	Water (5 °C/55 °C)	zirconia/primary enamel	1.426 ± 0.477 mm^3^	0.002 ± 0.001 mm^3^	Mild abrasion	No considerable features
				lithium disilicate/primary enamel	2.042 ± 0.696 mm^3^	0.006 ± 0.002 mm^3^	Abrasion	Microcracking/ abrasion
				leucite/primary enamel	2.670 ± 1.471 mm^3^	0.003 ± 0.002 mm^3^	Abrasion	Microcracking/ abrasion
				stainless steel/primary enamel	0.397 ± 0.192 mm^3^	0.002 ± 0.001 mm^3^	Abrasion	Plastic deformation and abrasion by ploughing
[55]	Pin-on-plate	9.8 N 1100 cycles 1.6 Hz Stroke: 0.2 mm	Distilled water	lithium disilicate/enamel	-	-	Abrasive wear	Adhered enamel layer
				type III gold/enamel	-	-	Adhesive wear	Polished surface
[56]	Chewing simulator	75 N 100,000 cycles 1.2 Hz	Distilled water	Dicor MGC Light /enamel	0.024 ± 0.014 mm^2^	0.153± 0.049 mm^2^	-	-
				Vita Mark II/enamel	0.078 ± 0.041 mm^2^	0.140 ± 0.02 mm^2^	-	-
				IPS Empress/enamel	0.089 ± 0.045 mm^2^	0.116 ± 0.038 mm^2^	-	-
				cast type III gold/enamel	0.019 ± 0.025 mm^2^	0.067 ± 0.036 mm^2^	-	-
[57]	Pin-on-disc	5 N 10,000 cycles	Human saliva	Olympia gold/enamel	9 ± 13 µm	0.32 ± 0.1 µm	-	-
				Procera All-Ceramic/enamel	60 ± 28 µm	4.3 ± 2.3 µm	-	-
				Ceramco feldspathic porcelain/enamel	230 ± 38 µm	3.7 ± 0.6 µm	-	-
[58]	Pin-on-disc	40 N 25,000 cycles Stroke: 10 mm	Distilled water	Alpha porcelain/enamel	0.93 ± 0.15 mm	76.04 ± 12.39 mm	-	-
				Omega porcelain/enamel	0.96 ± 0.20 mm	62.02 ± 20.85 mm	-	-
				Duceram-LFC/enamel	0.54 ± 0.15 mm	41.88 ± 17.36 mm	-	-
				Vita Mark II/enamel	0.65 ± 0.16 mm	25.86 ± 10.52 mm	-	-
				gold/enamel	0.09 ± 0.03 mm	16.28 ± 5.59 mm	-	-
[59]	Pin-on-plate	15 N 200,000 cycles 1 Hz	Water	zirconia/enamel	1 ± 0.2 µm	-	-	Adhesion of enamel particles
				stainless steel/enamel	0.6 ± 0.4 µm	-	-	Adhesion of enamel particles
[60]	Chewing simulator	49 N 200,000 cycles Stroke: 0.3 mm	Water	feldspathic ceramic/enamel	0.067 ± 0.018 mm^3^	-	-	Abrasion/ delamination
				nano-filled composite resin/enamel	0.016 ± 0.006 mm^3^	-	-	Pull-out
				acrylic resin/enamel	0.093 ± 0.021 mm^3^	-	-	Pull-out and fatigue wear
[61]	Pin-on-disc	9.8 N 100 rpm Stroke: 100 m	Distilled water	Lava Zirconia/enamel	~51 µm	-	-	No features
				Vintage MP veneering porcelain/enamel	~425 µm	-	-	Delamination
				Cerabien ZR veneering porcelain/enamel	~450 µm	-	-	Delamination
				Gradia Direct microhybrid composite resin/enamel	~85 µm	-	-	Microcracking (low extent) and scratching
				Filtek Z250 microhybrid composite resin/enamel	~165 µm	-	-	Microcracking between the filler and the matrix; particles pull-out
				Filtek Z350 nanocomposite/ enamel	~100 µm	-	-	Intensive plastic deformation with accumulation of resin particles
[62]	Chewing simulator	49 N 1,200,000 cycles 1.7 Hz	Water (5 °C/50 °C)	veneered zirconia (VZ)/enamel	73.5 ± 32.8 µm	66.8 ± 47.5 µm	Delamination	Delamination of the coating
				glazed zirconia (GZC)/enamel	118 ± 30.9 µm	49.5 ± 10.3 µm	-	Spalling of the coating
				glazed zirconia with glaze spray (GZS)/enamel	62.2 ± 16.6 µm	91.3 ± 38.6 µm	-	Spalling of the coating
				manually polished zirconia (MAZ)/enamel	27.3 ± 15.2 µm	0.8 ± 0.8 µm	-	No features
				mechanically polished zirconia (MEZ)/enamel	28 ± 11.1 µm	0.8 ± 0.8 µm	-	Abrasion
				monolithic base alloy (MA)/enamel	55.3 ± 38.5 µm	13.2 ± 8.3 µm	Polished surface	-
[63]	Chewing simulator	75 N 100,000 cycles 1.2 Hz	Water	microfilled composite (Epic-TMPT (Parkell))	0.5 × 10^−^^2^ mm^2^	4.5 × 10^−^^2^ mm^3^	-	-
				hybrid composite resin (Superlux Universal Hybrid (DMG))	0.8 × 10^−^^2^ mm^2^	4.5 × 10^−^^2^ mm^3^	-	-
				Clearfil AP-X (Kuraray Co.)	1.05 × 10^−^^2^ mm^2^	10 × 10^−^^2^ mm^3^	-	-
				Charisma (Kulzer Co.)	1.1 × 10^−^^2^ mm^2^	7 × 10^−^^2^ mm^3^	-	-
				Conquest Crystal (Jeneric/ Pentron Inc.)	1.1 × 10^−^^2^ mm^2^	6 × 10^−^^2^ mm^3^	-	-
				Estio LC (GC Co.)	1.2 × 10^−^^2^ mm^2^	7.5 × 10^−^^2^ mm^3^		
				Prisma TPH (L.D. Caulk Co.)	1 × 10^−^^2^ mm^2^	4.5 × 10^−^^2^ mm^3^	-	-
				Quartz-filled composite resin (Clearfil Photo Posterior (KurarayCo.)	4.05 × 10^−^^2^ mm^2^	11 × 10^−^^2^ mm^3^	-	-
				Zirconium silicate filled composite (Z100)	3.2 × 10^−^^2^ mm^2^	13.5 × 10^−^^2^ mm^3^	-	-
				Zirconium silicate filled composite (P-50)	5.1 × 10^−^^2^ mm^2^	17.5 × 10^−^^2^ mm^3^	-	-
				gold alloy	1.6 × 10^−^^2^ mm^2^	3 × 10^−^^2^ mm^3^	-	-
[64]	Chewing simulator	49 N	Water (5 °C–55 °C)	zirconia (Prettau)/enamel	0.04 ± 0.02 mm^3^	0.04 mm^3^	-	Dental particles transfer (?)
				zirconia (Lava)/enamel	0.04 ± 0.02 mm^3^	0.042 mm^3^	-	Dental particles transfer (?)
				zirconia (Rainbow)/enamel	0.04 ± 0.02 mm^3^	0.04 mm^3^	-	Dental particles transfer (?)
				lithium disilicate (e.max Press)/enamel	0.06 ± 0.03 mm^3^	0.08 mm^3^	-	Microfracture Dental particles transfer (?)
				low fusing porcelain (Vita-Omega 900)/enamel	0.11 ± 0.03 mm^3^	0.013 mm^3^	-	Microfracture Dental particles transfer (?)
[65]	Chewing simulator	50 N 360,000 cycles 1 Hz Stroke: 0.7 mm	Artificial saliva	zirconia (zirkonzahn)/enamel	6.4 ± 1.5 (×10^−^^5^) mm^3^/Nm	-	Abrasion, adhesive wear	Abrasion, adhesive wear
				glazed zirconia (zirkonzahn)/enamel	8.3 ± 1.2 (×10^−^^5^) mm^3^/Nm	0.5 ± 0.05 (×10^−^^5^) mm^3^/Nm	Abrasion, adhesive wear	Abrasion, adhesive wear, microfracture
[66]	Pin-on-disc	40 N 1500 revolutions 150 r/min	Natural Saliva (S) Food slurry (F)	fluorapatite/enamel	S: ~1.2 mm^3^	S: ~0.8 mm^3^	Abrasion, delamination, adhesive wear	Abrasion, delamination, adhesive wear
					F: ~0.01 mm^3^	F: ~0.01 mm^3^	Abrasion	Abrasion
				feldspar/enamel	S: ~1.25 mm^3^	S: ~1 mm^3^	Abrasion, delamination, adhesive wear,	Abrasion, delamination, adhesive wear
					F: ~0.01 mm^3^	F: ~0.01 mm^3^	Microcracking, abrasion	Abrasion

**Table 3:** Tribological in vitro studies concerning the wear at the occlusal restorative-restorative materials’ interface.

Ref.	Type of Test	Operational Conditions	Lubricant	Restorative Material	Counterbody	Restorative Material Wear	Restorative Material Wear Mechanisms
[90]	Ball-on-3-flat tribometer	30 N 25 rpm 1 h testing Stroke: 37 m	Artificial saliva	zirconia-Zpex (3Y-PSZ)	zirconia (3Y-TZP) ball	2.7 × 10^−^^6^ mm^3^/N·m	Abrasive wear
				zirconia-Zpex Smile (5Y-PSZ)		3.1 × 10^−^^6^ mm^3^/N·m	-
				zirconia-Zpex (graded)		3.3 × 10^−^^6^ mm^3^/N·m	-
				lithium disilicate (IPS e.max CAD)		1.2 × 10^−^^4^ mm^3^/N·m	Abrasive wear, microfracture
				feldspathic ceramic (Vitablocs)		5.5 × 10^−^^5^ mm^3^/N·m	-
				ceramic–polymer composites—Enamic		3.7 × 10^−^^5^ mm^3^/N·m	Abrasive wear, pull-out, fatigue
				ceramic–polymer composites—Lava Ultimate		7.7 × 10^−^^5^ mm^3^/N·m	-
[87]	Chewing simulator	49 N 120,000 cycles 1.6 Hz Stroke: 0.7 mm	Water	type III gold alloy	zirconia cusp	0.331 ± 0.138 mm^3^	-
				hot pressed ceramic (Imagine PressX)		0.508 ± 0.150 mm^3^	-
				hot pressed ceramic (IPS e.max Press)		0.459 ± 0.137 mm^3^	-
				CAD/CAM ceramic (IPS e.max CAD)		0.355 ± 0.133 mm^3^	-
				CAD/CAM ceramic (Celtra Duo)		0.542 ± 0.115 mm^3^	-
				CAD/CAM feldspathic porcelain (Vitablocs Mark II)		0.472 ± 0.133 mm^3^	-
[91]	Chewing simulator	200 N 200,000 cycles 1 Hz Stroke: 2 mm	33% glycerin solution	cross-linked PMMA (DCL)	zirconia cusp	17.3 ± 1.0 mm^3^	Abrasion, microfatigue (?)
				cross-linked acrylate polymer (ZCAD)		14.3 ± 0.8 mm^3^	Abrasion, microfatigue (?)
				cross-linked PMMA (TEL)		11.9 ± 2.0 mm^3^	Abrasion, microfatigue (?)
				nano-hybrid composite resin (PHO)		4.3 ± 1.0 mm^3^	Abrasion
[88]	Chewing simulator	49 N 120,000 cycles 1.6 Hz Stroke: 0.7 mm	Water	type III gold alloy (Aurocast8)	zirconia cusp	0.328 ± 0.140 mm^3^	-
				resin composite (Enamel plus HRi) light (L) and heat (H) cured		L: 1.452 ± 0.245 mm^3^ H: 1.016 ± 0.198 mm^3^	-
				resin composite (Filtek Supreme XTE) light (L) and heat (H) cured		L: 0.972 ± 0.247 mm^3^ H: 1.017 ± 0.239 mm^3^	-
				resin composite (Ceram.X duo) light (L) and heat (H) cured		L: 0.894 ± 0.259 mm^3^ H: 0.806 ± 0.397 mm^3^	-
				microhybrid resin composite (Enamel plus HRi-Function) light (L) and heat (H) cured		L: 0.529 ± 0.139 mm^3^ H: 0.464 ± 0.191 mm^3^	-
[93]	Chewing simulator	49 N 600,000 cycles 1.3 Hz Stroke: 0.3 mm	Water (5 °C–55 °C)	nanofilled composite resin	zirconia cusp	0.048 ± 0.017 mm^3^	Abrasion, delamination
				feldspathic ceramic		0.056 ± 0.008 mm^3^	Abrasion, microcracking
				nanofilled composite resin	alumina cusp	0.033 ± 0.013 mm^3^	Abrasion, delamination
				feldspathic ceramic		0.050 ± 0.018 mm^3^	Abrasion, microcracking
[94]	Ball-on-plate	30 N 1 Hz Stroke: 2 mm	Artificial saliva	zirconium-lithium silicate glass-ceramic	alumina ball	3.17 × 10^−^^5^ mm^3^/N·m	Abrasion, microcracking, thin and almost absent layer of debris
				polymer-infiltrated ceramic network		5.33 × 10^−^^5^ mm^3^/N·m	Thick and unstable tribolayer
[95]	Chewing simulator	50 N 360,000 cycles 1.2 Hz	Water	nanofilled composite resin (Filtek silorane)	alumina cusp	6.4 µm^3^	-
				microfilled composite resin (Ivoclar heliomolar)		3.1 µm^3^	-
				nanohybrid composite resin (Voco Grandio)		3.7 µm^3^	-
[64]	Chewing simulator	49 N	Water (5 °C–55 °C)	zirconia (Lava)	feldspathic porcelain cusp	0.027 mm^3^	-
				zirconia (Rainbow)		0.02 mm^3^	-
				lithium disilicate (e.max Press)		0.055 mm^3^	-
				low fusing porcelain (Vita-Omega 900)		0.028 mm^3^	-
[96]	Wear simulation device	78.5 N 1,200,000 cycles 2 Hz		Esthet X (EX)	stainless-steel cylinder	1.162 ± 0.139 mm^3^	-
				Filtek Supreme Plus (SP)		0.541 ± 0.072 mm^3^	-
				Filtek Z250 (Z2)		0.477 ± 0.044 mm^3^	-
				Tetric EvoCeram (EC)		0.584 ± 0.037 mm^3^	-
				Z100 Restorative (Z1)		0.248 ± 0.036 mm^3^	-
[97]	Wear simulator	250 000 cycles	Water	microhybrid composite (Filtek Z250)	CoCr alloy cusp	0.110 mm^3^	-
				type III gold alloy		0.021 mm^3^	-
				porcelain		0.006 mm^3^	-
[92]	Pin-on-disc	50 N 10,000 cycles 1.2 Hz Stroke: 3.7 mm	Water (W) 33% mass Poppy seeds (P) 30% mass PMMA beads (PMMA)	microfilled composite (Durafill)	zirconia ball	W: 0.1 mm^3^ P: 1.6 mm^3^ PMMA: 0.55 mm^3^	Abrasive wear, Microfatigue
				hybrid composite (Clearfil AP-X)		W: 1.25 mm^3^ P: 0.2 mm^3^ PMMA: 1.4 mm^3^	
				microhybrid composite (Filtek Z250)		W: 2.05 mm^3^ P: 0.15 mm^3^ PMMA: 0.5 mm^3^	
				nanofilled composite (Filtek Supreme XT)		W: 2.1 mm^3^ P: 0.15 mm^3^ PMMA: 0.4 mm^3^	
				nanohybrid composite (GC Kalore)		W: 0.15 mm^3^ P: 0.4 mm^3^ PMMA: 1.45 mm^3^	
				nanohybrid composite (MI flow)		W: 0.15 mm^3^ P: 0.5 mm^3^ PMMA: 1.2 mm^3^	
				nanohybrid composite (Venus Diamond)		W: 0.95 mm^3^ P: 0.35 mm^3^ PMMA: 2.05 mm^3^	
				nanohybrid composite (Venus Pearl)		W: 0.7 mm^3^ P: 0.15 mm^3^ PMMA: 2 mm^3^	

## Supplementary Materials

The following are available online at https://www.mdpi.com/1996-1944/13/16/3525/s1.
