# Supplementary material for: A State-of-the-Art Review on the Wear of the Occlusal Surfaces of Natural Teeth and Prosthetic Crowns
Source: Materials (Basel). 2020 Aug 10;13(16):3525. doi: 10.3390/ma13163525 (PMC7476047; doi:10.3390/ma13163525)
Supplement: Supplementary file 1 [file materials-13-03525-s001.pdf]

materials

Supplementary Materials

A State-of-the-Art Review on the Wear of the Occlusal Surfaces of Natural Teeth and Prosthetic Crowns

Teeth and saliva

Natural human teeth are constituted by an external crown and a root embedded in the jaw alveolar bone [4,12]. The superficial layer of the dental crown is enamel, the hardest tissue in the human body, which is constituted by 96% inorganic material (mainly hydroxyapatite ($Ca_{10}(PO_4)_6(OH)_2$) [99]), being the rest water (2–3%) and organic material (e.g., proteins such as amelogenin and enamelin) (1%–2%) [3,4]. Enamel presents a hierarchical structure: nanocrystals of hydroxyapatite (prisms with mean width 68 nm, thickness 25 nm and length 100–500 nm) are held together in parallel arrays by a thin protein-rich sheath forming rods with a hexagonal cross-section of average diameter of 3–5 µm that arrange perpendicularly to the crown surface [1,3,100]. Dentin underlies the enamel and surrounds the pulp chamber and root canals, being a hydrated biological composite, mineralized protein-rich, mostly made of phosphoric apatite crystallites. Contrarily to enamel, dentin presents a porous structure and is constituted by 70% inorganic material, 18% organic material, and 12% water [3,4]. It is considered to be elastic and soft which allows the whole tooth to resist impact forces that occur in the process of grinding and mastication [12]. Dentin-enamel junction is a biological interface between the enamel and dentin and presents a high fracture toughness (~5 to 10 times higher than enamel but ~75% lower than dentin) [101] and has the ability to dissipate stresses inhibiting further crack propagation [100,102]; together with the underlying dentin, supports the integrity of enamel by preventing its fracture during mastication processes [103,104]. The dental pulp is a soft connective tissue highly innervated and vascularized, which is in the core of the teeth, with a part within the crown (coronal pulp) and another between the cervix of the tooth and its apex, (radicular pulp) [5,105]. The pulp contributes to the dentin formation and is responsible for giving nutrients to the tooth [105,106].

Table S1. Density and main mechanical properties of enamel and dentin [4,107–109].

Tooth Parts	Hardness (GPa)	Fracture Toughness ($MPa.m^{1/2}$)	Modulus of Elasticity (GPa)	Tensile Strength (MPa)	Compressive Strength (MPa)	Flexural Strength (MPa)	Shear Modulus (GPa)	Density (g/cm^3)
**Enamel**	3–6	1.5	20–84.2	0.030–0.035	0.095–0.386	n/a	23–45	3
**Dentin**	0.9–1	1.5	10.2–15.6	0.04–0.276	0.249–0.315	200–212	6–11	2.2

Saliva consists in roughly 98% water and 2% organic and inorganic substances, including electrolytes (such as sodium, potassium, calcium, magnesium, bicarbonate, and phosphates), immunoglobulins, proteins, enzymes, and nitrogenous products, such as urea and ammonia [110,111], with a pH ranging between 5.3 and 7.8 for healthy individuals [112]. The presence of proteins like mucins, lactoferrin, and lysozyme, provides to saliva antibacterial properties, allowing to minimize/prevent the adhesion of microorganisms to oral tissue surfaces, thus contributing to the control of bacterial and fungal colonization [111,113]. Saliva also acts as a lubricant and therefore plays a protective role on the teeth surface, by reducing the effect of shear stresses on the occlusal surfaces, thus minimizing the friction between them and preventing the excessive wear rate of teeth [114–117]. Finally, saliva has an important role in the remineralization process of teeth because of the

high salivary concentrations of calcium and phosphate. Furthermore, it acts as a natural buffer, neutralizing acids, which impairs demineralization.

In pathological conditions, the amount of produced saliva may be lower than usual (xerostomia), impairing its normal functions (e.g., formation of food bolus, chemical digestion, lubrication, antibacterial effect). In particular, a low quantity of saliva can favor friction and wear of teeth [118]. According to Sun et al. [119], people who suffer from xerostomia revealed approximately ten-fold higher risk of tooth wear than people without this pathological condition.

Artificial Dental Materials Currently Used in Dentistry

A brief introduction to materials classes usually used for dental restorative applications is given below. Detailed reviews on this topic can be found in elsewhere [120–128].

Ceramics

Ceramics are a group of materials currently used in the dental field due to their attractive characteristics and properties which are similar to natural teeth, such as the high compressive strength, low thermal and electrical conductivity, radiopacity, color stability, and aesthetics [129]. The main applications of ceramic materials in dentistry are shown in Figure 1 and the respective mechanical properties in Table S2.

The translucency of the natural tooth may be mimicked using leucite, feldspar, and lithium disilicate [130]. However, there are concerns regarding their low fracture toughness [131,132]. Crystalline materials such as alumina and zirconia and their composites are more prone to be used in applications where the mechanical resistance is crucial (e.g., posterior restorations) [133–135].

The main issue of the ceramic materials is the lower toughness when compared to other materials. Several strategies have been investigated to enhance their mechanical resistance. For vitroceramics (leucite, lithium disilicate), the mechanical resistance is achieved by the control of the size and morphology of the crystalline phase within the vitreous matrix. The crystalline phase delays or suppresses the propagation of cracks through the absorption of the fracture energy, thus enhancing the toughness and strength of the material [136]. In addition, an increase of the mechanical resistance can be achieved by the reinforcement with zirconia particles [137–141].

In the case of alumina, this is achieved by a high control of the purity, porosity, and grain size [142]. The reduction of grain size increases the absorption of energy during the intergranular crack propagation impairing it. The reduction in the pore size leads to a decrease in the stress field minimizing the fracture possibility. A high purity is crucial to avoid superficial dissolution and stress concentration sites.

Concerning zirconia, the addition of stabilizing oxides (e.g., Y_2O_3 [143], leading to Yttria stabilized zirconia (Y-TZP)) allows keeping the metastable tetragonal at room temperature. The toughening mechanism for zirconia consists in the local transformation of the metastable tetragonal to stable monoclinic phase, induced by the crack stress field with a consequent volume expansion, impairing the crack growth [144].

The ceramic's mechanical properties can be reduced over time because of the ageing process. In fact, one of the drawbacks in the use of zirconia for dental applications is ageing, which consists in a progressive and spontaneous transformation of the metastable tetragonal phase to the monoclinic phase in wet and hot environment [145], as that found in oral cavity. This can be overcome by the addition of alumina to the zirconia matrix (alumina toughened zirconia (ATZ)) [146]. In turn, if alumina is reinforced with an amount of zirconia of approx. 18% (w/w) (zirconia toughened alumina (ZTA)), a toughness similar to zirconia is obtained.

Table S2. Density and main mechanical properties of ceramic materials used in dentistry [129,147–149].

Ceramics	Hardness (GPa)	Fracture Toughness (MPa.m $^{1/2}$)	Modulus of Elasticity (GPa)	Tensile Strength (MPa)	Compressive Strength (MPa)	Flexural Strength (MPa)	Shear Modulus (GPa)	Density (g/cm^3)
**Zirconia (ZrO_2)**	5–15	1–8	100–250	115–711	1200–5200	177–1000	54.4–86.4	5.68
**Alumina (Al_2O_3)**	20–22	3.3–5	380	69–665	690–5500	500	88–165	3.95
**Leucite ($KAlSi_2O_6$)**	5.3–7.9	0.8–1.3	65–67	n/a	n/a	55–134	n/a	2.44
**Lithium disilicate ($Li_2Si_2O_5$)**	5.5–6.8	2–3.5	90–100	n/a	n/a	250–365	50.12	2.4

Resin-Based Restorative Materials

Resin based restorative materials are aesthetically attractive, presenting translucency and color that match a variety of shades of natural teeth. They can easily bond to enamel and dentin. UV-cured acrylic resins are commonly used in dentistry in several areas such as restorative, prosthetics, endodontics, and orthodontics [126]. Acrylic resins are generally based on the esters of methacrylic acid, being the most common monomer methyl methacrylate [150]. Restorative resins are commonly composed of bisphenol A-glycidyl methacrylate (BIS-GMA) and other methacrylate monomers such as urethane dimethacrylate (UDMA), triethylene glycol dimethacrylates (TEGDMA), and bisphenol A-polyethylene glycol diether dimethacrylate (Bis-EMA)), and contain a photoinitiator (e.g., benzoyl peroxide) [151]. These resins are used for sealants that aim to restore in situ teeth appearance, structure and/or function. However, this type of resins present some drawbacks such as high wear rate, low mechanical properties, and high volume shrinkage during polymerization [152]. The latter leads to the formation of microgaps between the resin restorative material and the tooth, which can induce the formation of secondary caries [153,154]. In order to improve the mechanical properties and reduce the shrinkage, fillers (e.g., silica, glass and glass–ceramic particles [155]) can be added in different amounts, giving rise to different types of composite resins [156]. Usually, silane coupling agents are used to coat the filler particles in order to improve the interface bonding between the filler particles and the methacrylate network [157]. These composites are classified according to the size of the filler reinforcing particles as macrofilled (traditional) composites, microfilled composites, hybrid composites, and nanofilled composites [153,154,158]. Table S3 summarizes some relevant properties of different types of restorative resins.

Table S3. Main properties of resin-based materials used in dentistry [126,159,160].

Resin-based Materials	Particle Size	Filler Amount (% wt.)	Modulus of Elasticity (GPa)	Tensile Strength (MPa)	Compressive Strength (MPa)	Coefficient of Thermal Expansion (10^{-6}/ °C)
**Unfilled resin**	-	-	2.4	24	70	92.8
**Macrofilled composites**	10–50 μm	70–80	8–15	50–65	250–300	25–35
**Microfilled composites**	40–50 nm	~60%	3–6	30–50	250–350	50–60

**Hybrid composites**	10–50 μm + 40 nm	70–80%	7–12	70–90	300–350	30–40
**Nanofilled composites**	5–100 nm	~75%	12–20	30–46	291–299	n/a

Metals and Alloys

Metals and their alloys present suitable mechanical properties (e.g., low hardness, high toughness) to be used in dentistry. However, these materials are not the main choice for several applications, mainly because of aesthetic reasons. The currently used metal materials are gold-base alloys, amalgams, stainless steel, nickel–chromium, and cobalt–chromium alloys (Table S4). Gold alloys and amalgams are usually used in dental obturation, while nickel–chromium and cobalt–chromium alloys for porcelain fused to metal (PFM) dentures. Stainless steel is mainly used in dental copings in first teeth for children [4,161,162]. Corrosion of non-noble alloys is one of the main issues concerning the use of metals, mainly for some amalgams where bleaching of mercury may occur [163].

Table S4. Density and main mechanical properties of metallic materials used in dentistry [127,128,161,164–171].

Metals	Hardness (GPa)	Modulus of Elasticity (GPa)	Tensile Strength (MPa)	Yield Strength (MPa)	Shear Strength (MPa)	Elongation (%)	Density (g/cm³)
**Gold alloy**	0.25–3.04	95–123	410–770	420–575	185–276	6.5–45	13.5–19.3
**Amalgam**	0.88	28–29	32	n/a	n/a	n/a	13.75–14.1
**Stainless steel**	1.402–3.04	134–205	2035–2849	965–1680	74.5–597	2–3.2	8
**Ni–Cr alloys**	3.521–4.139	141–248	539–919	180–858	n/a	<1–32.6	7.9–8.7
**Co–Cr–Mo alloys**	2.5–3.187	155–240	655–889	390–644	116.5	1.5–10	8.5

References

1. Zhang, Y.; Du, W.; Zhou, X.; Yu, H. Review of research on the mechanical properties of the human tooth. *Int. J. Oral Sci.* **2014**, *6*, 61–69, doi:10.1038/ijos.2014.21.
3. Roy, S.; Basu, B. Mechanical and tribological characterization of human tooth. *Mater. Charact.* **2008**, *59*, 747–756, doi:10.1016/j.matchar.2007.06.008.
4. Zhou, Z.R.; Zheng, J. Tribology of dental materials: A review. *J. Phys. D Appl. Phys.* **2008**, *41*, 11301.
5. Chen, J. Food oral processing—A review. *Food Hydrocoll.* **2009**, *23*, 1–25, doi:10.1016/j.foodhyd.2007.11.013.
12. Lewis, R.; Dwyer-Joyce, R.S. Wear of human teeth: A tribological perspective. *Proc. Inst. Mech. Eng. Part J J. Eng. Tribol.* **2005**, *219*, 2–19, doi:10.1243/135065005X9655.
99. Mair, L.H.; Padipatvuthikul, P. Wear mechanisms in the mouth. *Proc. Inst. Mech. Eng. Part J J. Eng. Tribol.* **2010**, 569–575, doi:10.1243/13506501JET686.
100. Biswas, N.; Dey, A.; Kundu, S.; Chakraborty, H.; Mukhopadhyay, A.K. Mechanical Properties of Enamel Nanocomposite. *ISRN Biomater*. **2013**, *2013*, 1–15.
101. Imbeni, V.; Kruzic, J.J.; Marshall, G.W.; Marshall, S.J.; Ritchie, R.O. The dentin–enamel junction and the fracture of human teeth. *Nat. Mater.* **2005**, *4*, 229–232, doi:10.1038/nmat1323.
102. Low, I.M.; Duraman, N.; Mahmood, U. Mapping the structure, composition and mechanical properties of human teeth. *Mater. Sci. Eng. C* **2008**, *28*, 243–247, doi:10.1016/j.msec.2006.12.013.
103. Cuy, J.L.; Mann, A.B.; Livi, K.J.; Teaford, M.F.; Weihs, T.P. Nanoindentation mapping of the mechanical properties of human molar tooth enamel. *Arch. Oral Biol.* **2002**, *47*, 281–291.

104. Xul, H.H.K.; Smith, D.T.; Jahanmir, S.; Romberg, E.; Kelly, J.R.; Thompson, V.P.; Rekow, E.D. Indentation Damage and Mechanical Properties of Human Enamel and Dentin. *J. Dent. Res.* **1998**, *77*, 472–480, doi:10.1177/00220345980770030601.
105. Andersson, L.; Kahnberg, K.-E.; Pogrel, M.A. *Oral and Maxillofacial Surgery*; Wiley-Blackwell: Hoboken, NJ, USA, 2010.
106. Yu, C.; Abbott, P.V. An overview of the dental pulp: Its functions and responses to injury. *Aust. Dent. J. Suppl.* **2007**, *52*, 4–16.
107. Yadav, S.; Gangwar, S. A critical evaluation of tribological interaction for restorative materials in dentistry. *Int. J. Polym. Mater. Polym. Biomater.* **2018**, 1005–1019, doi:10.1080/00914037.2018.1525544.
108. Arikawa, H. Dynamic shear modulus in torsion of human dentin and enamel. *Dent. Mater. J.* **1989**, *8*, 223–235.
109. Ratih, D.N. Influence of Exposing Root Canal Dentin to Calcium Hydroxide on Its Flexural Strength. *Indones. J. Dent. Res.* **2015**, *1*, 143, doi:10.22146/theindjdentres.10063.
110. Aguirre, A.; Tabak, L.A.; Dysfunction, S.G. Artificial Salivas: Present and Future. *J. Dent. Res.* **1987**, *66*, 693–698.
111. Humphrey, S.P.; Williamson, R.T. A review of saliva: Normal composition, flow, and function. *J. Prosthet. Dent.* **2001**, *85*, 162–169.
112. Khemiss, M.; Ben Khelifa, M.; Ben Saad, H. Preliminary findings on the correlation of saliva pH, buffering capacity, flow rate and consistency in relation to waterpipe tobacco smoking. *Libyan J. Med.* **2017**, *12*, doi:10.1080/19932820.2017.1289651.
113. Bodiba, D.; Szuman, K.M.; Lall, N. Chapter 6—The Role of Medicinal Plants in Oral Care. In *Medicinal Plants for Holistic Health and Well-Being*; Academic Press: Cambridge, MA, USA, 2018; pp. 183–212.
114. Ranc, H.; Elkhyat, A.; Servais, C.; Mac-Mary, S.; Launay, B.; Humbert, P. Friction coefficient and wettability of oral mucosal tissue: Changes induced by a salivary layer. *Colloids Surf.* **2006**, *276*, 155–161, doi:10.1016/j.colsurfa.2005.10.033.
115. Ranc, H.; Servais, C.; Chauvy, P.; Debaud, S.; Mischler, S. Effect of surface structure on frictional behaviour of a tongue/palate tribological system. *Tribol. Int.* **2006**, *39*, 1518–1526, doi:10.1016/j.triboint.2006.01.017.
116. Prinz, J.F.; Wijk RADe Huntjens, L. Load dependency of the coefficient of friction of oral mucosa. *Food Hydrocoll.* **2007**, *21*, 402–408, doi:10.1016/j.foodhyd.2006.05.005.
117. Sajewicz, E. Effect of saliva viscosity on tribological behaviour of tooth enamel. *Tribol. Int.* **2009**, *42*, 327–332, doi:10.1016/j.triboint.2008.07.001.
118. Young, W.G. The oral medicine of tooth wear. *Aust. Dent. J.* **2001**, *46*, 236–250, doi:10.1111/j.1834-7819.2001.tb00288.x.
119. Sun, K.; Wang, W.; Wang, X.; Shi, X.; Si, Y.; Zheng, S. Tooth wear: A cross-sectional investigation of the prevalence and risk factors in Beijing, China. *BDJ Open* **2017**, *3*, 1–7, doi:10.1038/bdjopen.2016.12.
120. Fu, L.; Engqvist, H.; Xia, W. Glass-Ceramics in Dentistry: A Review. *Materials* **2020**, *13*, E1049.
121. Sulaiman, T.A. Materials in digital dentistry—A review. *J. Esthet. Restor. Dent.* **2020**, *32*, 171–181, doi:10.1111/jerd.12566.
122. Grech, J.; Antunes, E. Zirconia in dental prosthetics: A literature review. *J. Mater. Res. Technol.* **2019**, *8*, 4956–4964, doi:10.1016/j.jmrt.2019.06.043.
123. Zarone, F.; Di Mauro, M.I.; Ausiello, P.; Ruggiero, G.; Sorrentino, R. Current status on lithium disilicate and zirconia: A narrative review. *BMC Oral Health* **2019**, *19*, 1–14, doi:10.1186/s12903-019-0838-x.
124. Kaur, M.; Singh, K. Review on titanium and titanium based alloys as biomaterials for orthopaedic applications. *Mater. Sci. Eng. C* **2019**, *102*, 844–862, doi:10.1016/j.msec.2019.04.064.
125. Yadav, R.; Kumar, M. Dental restorative composite materials: A review. *J. Oral Biosci.* **2019**, *61*, 78–83, doi:10.1016/j.job.2019.04.001.
126. Burrow, M.F. Composite adhesive restorative materials for dental applications. In *Non-Metallic Biomater. Tooth Repair Replace*; Woodhead Publishing: Sawston, UK, 2013; pp. 235–269, doi:10.1533/9780857096432.3.235.
127. Sakaguchi, R.L.; Powers, J.M. *Craig's Restorative Dental Materials*, 13th ed.; Elsevier/Mosby: Philadelphia, PA, USA, 2011.
128. Roach, M. Base Metal Alloys Used for Dental Restorations and Implants. *Dent. Clin. N. Am.* **2007**, *51*, 603–627, doi:10.1016/j.cden.2007.04.001.

129. Galante, R.; Figueiredo-Pina, C.G.; Serro, A.P. Additive manufacturing of ceramics for dental applications: A review. *Dent. Mater.* **2019**, doi:10.1016/j.dental.2019.02.026.
130. Bajraktarova-Valjakova, E.; Korunoska-Stevkovska, V.; Kapusevska, B.; Gigovski, N.; Grozdanov, A.Contemporary Dental Ceramic Materials, A Review: Chemical Composition, Physical and Mechanical Properties, Indications for Use. *Maced. J. Med. Sci.* **2018**, *6*, 1742–1755.
131. Mizrahi, B. All-ceramic silica/glass-based crowns—Clinical protocols. *Br. Dent. J.* **2011**, *211*, 257–262, doi:10.1038/sj.bdj.2011.767.
132. Saint-Jean, S.J. Chapter 12—Dental Glasses and Glass-ceramics. In *Advanced Ceramics for Dentistry*; Butterworth-Heinemann: Oxford, UK, 2014; pp. 255–277.
133. Alfawaz, Y. Zirconia crown as single unit tooth restoration: A literature review. *J. Contemp. Dent. Pract.* **2016**, *17*, 418–422, doi:10.5005/jp-journals-10024-1865.
134. Tang, Z.; Zhao, X.; Wang, H.; Liu, B. Clinical evaluation of monolithic zirconia crowns for posterior teeth restorations. *Medicine* **2019**, *98*, doi:10.1097/MD.0000000000017385.
135. Al-Sanabani, F.A.; Madfa, A.A.; Al-Qudaimi, N.H. Alumina ceramic for dental applications: A review article. *Am. J. Mater. Res.* **2014**, *1*, 26–34.
136. Serbena, F.C.; Mathias, I.; Foerster, C.E.; Zanotto, E.D. Crystallization toughening of a model glass-ceramic. *Acta Mater.* **2015**, *86*, 216–228, doi:10.1016/j.actamat.2014.12.007.
137. Santos, R.L.P.; Buciumeanu, M.; Silva, F.S.; Souza, J.C.M.; Nascimento, R.M.; Motta, F.V. Tribological behavior of zirconia-reinforced glass–ceramic composites in artificial saliva. *Tribol. Int.* **2016**, *103*, 379–387, doi:10.1016/j.triboint.2016.07.019.
138. Santos, R.L.P.; Silva, F.S.; Nascimento, R.M.; Motta, F.V.; Souza, J.C.M.; Henriques, B. On the mechanical properties and microstructure of zirconia-reinforced feldspar-based porcelain. *Ceram. Int.* **2016**, *42*, 14214–14221, doi:10.1016/j.ceramint.2016.05.195.
139. Elsaka, S.E.; Elnaghy, A.M. Mechanical properties of zirconia reinforced lithium silicate glass-ceramic. *Dent. Mater.* **2016**, *32*, 908–914, doi:10.1016/j.dental.2016.03.013.
140. Tamayo, J.G.; Arango, A.O.R.; Henao, E.A.O. Improving the mechanical properties of commercial feldspathic dental porcelain by addition of Alumina-Zirconia. *Rev. Fac. Ing.* **2020**, 67–76, doi:10.17533/udea.redin.n91a11.
141. Santos, R.L.P.; Buciumeanu, M.; Silva, F.S.; Souza, J.C.M.; Nascimento, R.M.; Motta, F.V. Tribological behaviour of glass-ceramics reinforced by Yttria Stabilized Zirconia. *Tribol. Int.* **2016**, *102*, 361–370, doi:10.1016/j.triboint.2016.05.047.
142. Dearnley, P.A. A review of metallic, ceramic and surface-treated metals used for bearing surfaces in human joint replacements. *Proc. Inst. Mech. Eng. Part H J. Eng. Med.* **1999**, *213*, 107–135, doi:10.1243/0954411991534843.
143. Denry, I.; Holloway, J.A. Ceramics for Dental Applications: A Review. *Materials* **2010**, *3*, 351–368, doi:10.3390/ma3010351.
144. Denry, I.; Kelly, J.R. State of the art of zirconia for dental applications. *Dent. Mater.* **2008**, *24*, 299–307, doi:10.1016/j.dental.2007.05.007.
145. Guo, X. Property degradation of tetragonal zirconia induced by low-temperature defect reaction with water molecules. *Chem. Mater.* **2004**, *16*, 3988–3994, doi:10.1021/cm040167h.
146. Pabst, W.; Havrda, J.I.Ř.Í.; Gregorová, E.V.A.; Mová, B.K.R.Č. Alumina toughened zirconia made by room temperature extrusion of ceramic pastes. *Ceramics* **2000**, *44*, 41–47.
147. AZO Materials. Zirconia—ZrO2, Zirconium Dioxide N.D. Available online: https://www.azom.com/properties.aspx?ArticleID=133 (accessed on 23 July 2020).
148. AZO Materials. Alumina—Aluminium Oxide—Al2O3—A Refractory Ceramic Oxide N.D. Available online: https://www.azom.com/properties.aspx?ArticleID=52 (accessed on 23 July 2020).
149. Biskri, Z.E.; Rached, H.; Bouchear, M.; Rached, D.; Aida, M.S. A Comparative Study of Structural Stability and Mechanical and Optical Properties of Fluorapatite (Ca5(PO4)3F) and Lithium Disilicate (Li2Si2O5) Components Forming Dental Glass–Ceramics: First Principles Study. *J. Electron. Mater.* **2016**, *45*, 5082–5095, doi:10.1007/s11664-016-4681-4.
150. Sastri, V.R. Acrylics. In *Plastics in Medical Devices*; William Andrew: Burlington, MA, USA, 2000.
151. Silver, F. Dental implants. In *Biomaterials, Medical Devices and Tissue Engineering: An Integrated Approach*; Springer: Dordrecht, The Netherlands, 1994.

152. Alshaafi, M.M. Factors affecting polymerization of resin-based composites: A literature review. *Saudi Dent. J.* **2017**, *29*, 48–58, doi:10.1016/j.sdentj.2017.01.002.
153. Nazhat, S.N. Composites for dental applications. *Biomed. Compos.* **2010**, 201–209, doi:10.1533/9781845697372.2.201.
154. Hamouda, I.M.; Elkader, H.A. Evaluation the Mechanical Properties of Nanofilled Composite Resin Restorative Material. *J. Biomater. Nanobiotechnol.* **2012**, *3*, 238–242.
155. Heintze, S.D.; Zellweger, G.; Zappini, G. The relationship between physical parameters and wear of dental composites. *Wear* **2007**, *263*, 1138–1146, doi:10.1016/j.wear.2006.12.010.
156. Van Foreest, A.W. Adhesive Dentistry with Direct Restorative Materials. *Vet. Q.* **2014**, *2176*, 18–20, doi:10.1080/01652176.1998.10807396.
157. Tarle Z, Par M. Bioactive dental composite materials. *Med. Sci.* **2018**, *45*, 83–100.
158. Ferracane, J.L. Resin composite—State of the art. *Dent. Mater.* **2010**, *27*, 29–38, doi:10.1016/j.dental.2010.10.020.
159. Alzraikat, H.; Burrow, M.; Maghaireh, G.; Taha, N. Nanofilled Resin Composite Properties and Clinical Performance: A Review. *Oper Dent*. **2018**, *43*, E173–E190, doi:10.2341/17-208-T.
160. Physics, L.; Rabelo, S.; Universidade, C.; Grande, R.; Calabrez-Filho, S.; Campos, E.A. Compressive Strength of Dental Composite Resins Photo-Activated with Different Light Compressive strength of dental composites photo-activated with different light tips. *Laser Phys*. **2013**, *23*, doi:10.1088/1054-660X/23/4/045604.
161. Slokar, L.; Pranjić, J.; Carek, A. Metallic materials for use in dentistry. *Holist Approach Environ.* **2017**, *7*, 39–58.
162. Abdolahpour, Z.; Saneipour, Z.; Azarhoosh, M.J. Application of Biomaterials in Dentistry. *Curr. Trends Biomed. Eng. Biosci.* **2017**, *2*, 8–11, doi:10.19080/CTBEB.2016.01.555588.
163. Brett, C.M.A.; Trandafir, F. The corrosion of dental amalgam in artificial salivas: An electrochemical impedance study. *J. Electroanal. Chem.* **2004**, *572*, 347–354, doi:10.1016/j.jelechem.2004.01.003.
164. Osman, R.B.; Swain, M.V. A critical review of dental implant materials with an emphasis on titanium versus zirconia. *Materials* **2015**, *8*, 932–958, doi:10.3390/ma8030932.
165. Souza, J.C.M.; Henriques, M.; Teughels, W.; Ponthiaux, P.; Celis, J.P.; Rocha, L.A. Wear and Corrosion Interactions on Titanium in Oral Environment: Literature Review. *J Biol. Tribol. Corros.* **2015**, *1*, doi:10.1007/s40735-015-0013-0.
166. Chun, K.J.; Lee, J.Y. Comparative study of mechanical properties of dental restorative materials and dental hard tissues in compressive loads. *J. Dent. Biomech.* **2014**, *5*, 1–6, doi:10.1177/1758736014555246.
167. The European Stainless Steel Development Association (Euro Inox). *Stainless Steel: Tables of Technical Properties*; The European Stainless Steel Development Association (Euro Inox): Madrid, Spain, 2007; Volume 5.
168. Overview of Materials for Stainless Steel N.D. Available online: http://www.matweb.com/search/datasheet_print.aspx?matguid=71396e57ff5940b791ece120e4d563e0 (accessed on 25 July 2020).
169. De Oliveira Bauer, J.R.; Loguercio, A.D.; Reis, A.; Filho, L.E.R. Microhardness of Ni-Cr alloys under different casting conditions. *Braz. Oral Res.* **2006**, *20*, 40–46, doi:10.1590/s1806-83242006000100008.
170. Meacock, C.G.; Vilar, R. Structure and properties of a biomedical Co-Cr-Mo alloy produced by laser powder microdeposition. *J. Laser Appl.* **2009**, *21*, 88–95, doi:10.2351/1.3120214.
171. Henriques, B.; Soares, D.; Silva, F.S. Microstructure, hardness, corrosion resistance and porcelain shear bond strength comparison between cast and hot pressed CoCrMo alloy for metal-ceramic dental restorations. *J. Mech. Behav. Biomed. Mater.* **2012**, *12*, 83–92, doi:10.1016/j.jmbbm.2012.03.015.

© 2020 by the authors. Submitted for possible open access publication under the terms and conditions of the Creative Commons Attribution (CC BY) license (http://creativecommons.org/licenses/by/4.0/).
